# An Approach to Improve SSD through Skip Connection of Multiscale Feature Maps

**DOI:** 10.1155/2020/2936920

**Published:** 2020-04-05

**Authors:** Xiaoguo Zhang, Ye Gao, Fei Ye, Qihan Liu, Kaixin Zhang

**Affiliations:** School of Instrument Science and Engineering, Southeast University, Nanjing 210096, China

## Abstract

SSD (Single Shot MultiBox Detector) is one of the best object detection algorithms and is able to provide high accurate object detection performance in real time. However, SSD shows relatively poor performance on small object detection because its shallow prediction layer, which is responsible for detecting small objects, lacks enough semantic information. To overcome this problem, SKIPSSD, an improved SSD with a novel skip connection of multiscale feature maps, is proposed in this paper to enhance the semantic information and the details of the prediction layers through skippingly fusing high-level and low-level feature maps. For the detail of the fusion methods, we design two feature fusion modules and multiple fusion strategies to improve the SSD detector's sensitivity and perception ability. Experimental results on the PASCAL VOC2007 test set demonstrate that SKIPSSD significantly improves the detection performance and outperforms lots of state-of-the-art object detectors. With an input size of 300 × 300, SKIPSSD achieves 79.0% mAP (mean average precision) at 38.7 FPS (frame per second) on a single 1080 GPU, 1.8% higher than the mAP of SSD while still keeping the real-time detection speed.

## 1. Introduction

The object detection algorithms based on deep learning could be roughly divided into two categories: based on region proposals and based on end to end. As known, the former models generally include R-CNN [1], Fast R-CNN [2], Faster R-CNN [3], and R-FCN [4], which firstly generate a category-independent set of region proposals for subsequent feature extraction and classification. The two most popular latter models based on end to end are YOLO (You Only Look Once) [5] and SSD (Single Shot MultiBox Detector) [6], which need setting the default box, training the network, and establishing the relationship of the prior box, default box, and ground truth box.

**Figure 1:**
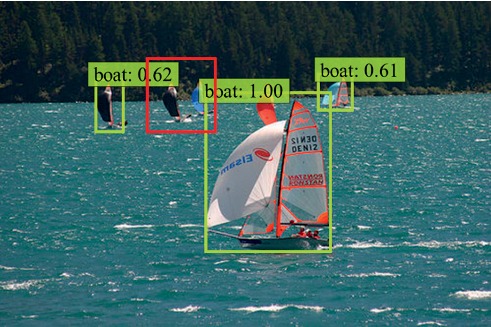
The framework of our SKIPSSD. SKIPSSD skippingly fuses high-level and low-level feature maps to enhance semantic information of the model.

The two-stage methods such as SPP-net [7], Fast R-CNN [2], and Faster R-CNN [3], generally only use the last layer as the prediction layer, but the layer with the fixed receptive field size is not suitable for both too larger and smaller object detections. SSD innovatively uses the pyramid feature hierarchy of ConvNet and combines predictions from multiple feature maps with different resolutions to deal with the scale variation problem for object detector. Generally speaking, SSD is not only able to achieve real-time object detection but also known for its high detection accuracy. On the PASCAL VOC 2007 test [8], SSD achieves 77.2% mAP at the speed of 46 FPS with the input size 300 × 300 using a single NVIDIA Titan X GPU [6]. However, the linkages between multiscale prediction layers of SSD are not fully considered, and the low-level feature maps lack enough semantic information for small object detection; thus, SSD shows poor performance on small object detection [9]. As shown in Figure 1, some small objects, for example boats in the red box, are not detected by SSD.

**Figure 2:**
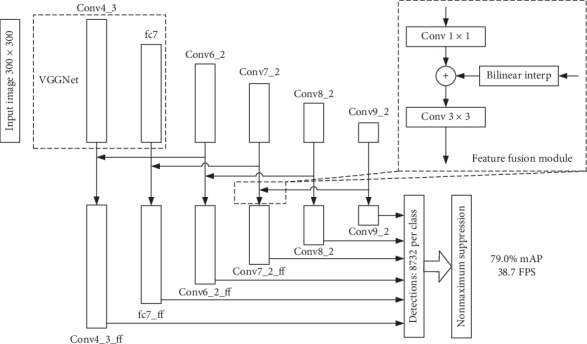
The framework of our SKIPSSD. SKIPSSD skippingly fuses high-level and low-level feature maps to enhance semantic information of the model.

To deal with the problem that SSD shows poor performance on small object detection and to maintain a satisfactory detection speed at the same time, we adopt a novel skip connection of multiscale feature maps to SSD, and the overall architecture is illustrated in Figure 2. The main contributions are summarized as follows: (1) SKIPSSD, an improved SSD with a novel skip connection of multiscale feature maps, is proposed to enhance the semantic information and the details of the prediction layers through skippingly fusing high-level and low-level features; (2) six multiscale feature maps fusion structures over the SSD network, and two feature fusion modules and multiple fusion strategies are designed to investigate the optimal feature fusion framework; (3) experiments on the PASCAL VOC 2007 test set are conducted to compare the performance of SKIPSSD with other state-of-the-art object detectors.

The experimental results demonstrate that SKIPSSD significantly improves the detection performance and outperforms a lot of state-of-the-art object detectors. With an input size of 300 × 300, SKIPSSD achieves 79.0% mAP (mean average precision) at 38.7 FPS (frame per second) on a single 1080 GPU, 1.8% higher than the mAP of SSD while still keeping the real-time detection speed.

## 2. Methodology

### 2.1. Related Work

In the field of object detection, image pyramids are often used to solve the degradation of detection performance caused by the change of object scale. However, such kind of algorithms is very time consuming. SSD innovatively uses a ConvNet's pyramidal feature hierarchy and combines predictions from multiple layers with different scales, mitigating the problem of object scale change in certain degree [6]. However, the linkages between multiscale prediction layers of SSD are not fully considered, and the low-level feature maps lack enough semantic information for small object detection; thus, SSD shows poor performance on small object detection [9].

In order to deal with the abovementioned problem of SSD, DSSD (Deconvolutional Single Shot Detector) [10] uses Resnet-101 [11] in place of VGG used in SSD and adds deconvolutional layers to introduce large-scale context. Although DSSD improves the performance of small object detection of SSD, its detection speed is much slower than SSD, and it is not able to realize real-time detection. After that, much has been done to balance accuracy and speed for small object detection of SSD. RSSD [12] adopts weight-sharing strategy between different layers to SSD and improves the accuracy by 0.8% with the speed dropping to 35 FPS because of the increase of computational complexity. FSSD [9] uses a lightweight and efficient feature fusion module and achieves 78.8% mAP on the VOC2007 test set at 65.8 FPS, outperforming RSSD300 on both accuracy and speed. Feature-Fused SSD300 [13] simply conducts elt_sum function between Conv4_3 and Conv5_3 of SSD and achieves 78.9% mAP, which is little higher than FSSD. Combining the advantages of the two-stage and one-stage methods, RefineDet [14] uses the ARM module to reduce the search space, transfers the features from the ARM to ODM module through the TCB connection module, and achieves 81.8% mAP at 40.3 FPS with an input size 512 × 512, surpassing DSSD513. To further improve the accuracy of SSD, DES (Detection with Enriched Semantics) [15] not only introduces the segmentation module to obtain the mask, which objectively enhances the semantic information of the shallow features, but also introduces the global activation module to enhance the semantic information of the high-level features. DES512 boosts the mAP on the VOC2007 test set to 81.7% at 31.7 FPS. The accuracy of DES512 is improved at the cost of speed.

The abovementioned algorithms improve the detection accuracy of SSD. However, their inference time increase a lot. Overall speaking, it is of great value to obtain high precision object detection performance with satisfactory real time performance.

### 2.2. Multiscale Feature Fusion Structure Design

Many works have led to the discovery that the features from different layers of the network are complementary, and integrating the multiscale features can benefit the multiscale object detection [9, 10, 16]. More specifically, the very invariance properties of the DCNN model make the high-level feature maps of DCNN learn abstract features of data well which is good for object recognition but show side effect to object location, and the low-level feature maps contain rich spatial structural details which are beneficial to locate objects. Inspired by an encoder-decoder network U-Net [17], which uses the skip connection to associate low-level feature maps and high-level feature maps to realize the positioning at the pixel level, we adopt the idea of skip connection to SSD to enhance semantic information. In this paper, we design and evaluate a series of multiscale feature maps fusion structures over SSD to explore the optimal fusion structure: multiscale prediction feature maps skip connection (SKIPSSD), part skip connection (Part-SKIPSSD), bidirectional skip connection (Bi-SKIPSSD), skip connection with partial feature maps of base network (Base-SKIPSSD), adjacent connection (AdjacentSSD), and multiscale prediction feature pyramid network (FPNSSD).

**Figure 3:**
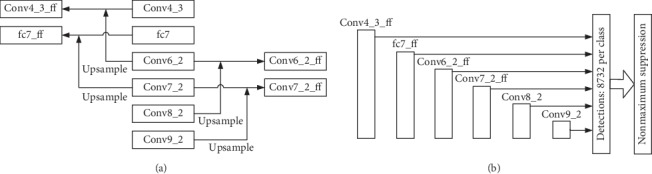
Multiscale prediction feature maps skip connection (SKIPSSD). (a) Skip connection structure. (b) Multiscale prediction layers.

Figure 3(a) shows the skip connection between the multiscale prediction feature maps, where SKIPSSD upsamples Conv9_2 to fuse with Conv7_2 to get Conv7_2_ff, upsamples Conv8_2 to fuse with Conv6_2 to get Conv6_2_ff, upsamples Conv7_2 to fuse with fc7 to get fc7_ff, and upsamples Conv6_2 to fuse with Conv4_3 to get Conv4_3_ff. The multiscale prediction feature maps skip-connection network is called SKIPSSD in this paper.  Figure 3(b) shows the structure of the prediction layer of SKIPSSD, Conv8_2, and Conv9_2, and the fused feature maps Conv4_3_ff, fc7_ff, Conv6_2_ff, and Conv7_2_ff are used as the multiscale prediction layers.

**Figure 4:**
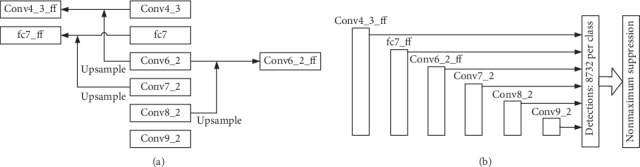
Multiscale prediction feature maps part skip connection (part-SKIPSSD). (a) Part skip connection structure. (b) Multiscale prediction layers.

As shown in Figure 4(a), the structure of Part-SKIPSSD is described. Compared with SKIPSSD, Part-SKIPSSD removes the feature fusion of Conv7_2 layer and only uses the Conv4_3_ff, fc7_ff, and Conv6_2_ff and Conv7_2, Conv8_2, and Conv9_2 of the original SSD as the multiscale prediction feature maps.

**Figure 5:**
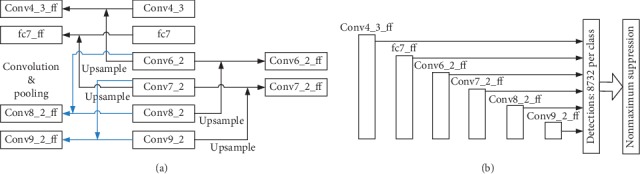
Multiscale prediction feature maps bidirectional skip connection (Bi-SKIPSSD). (a) Bidirectional skip connection structure. (b) Multiscale prediction layers.

The structure in Figure 5(a) is called Bi-SKIPSSD in this paper. Compared with SKIPSSD, Bi-SKIPSSD adds the feature fusion of layers Conv8_2 and Conv9_2, respectively. Conv6_2, which undergoes convolution and pooling layers, is then merged with Conv8_2 to generate Conv8_2_ff. Similarly, Conv7_2, which undergoes convolution and pooling layers, is then merged with Conv9_2 to generate Conv9_2_ff. The Figure 5(b) shows that Bi-SKIPSSD uses Conv4_3_ff, fc7_ff, Conv6_2_ff, Conv7_2_ff, Conv8_2_ff and Conv9_2_ff as the multiscale prediction feature maps.

**Figure 6:**
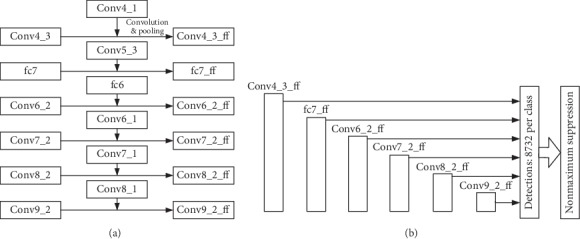
Skip connection with partial feature maps of base network (base-SKIPSSD). (a) Skip connection structure with partial feature maps of base network. (b) Multiscale prediction layers.

Different from the abovementioned three networks, Base-SKIPSSD in Figure 6 conducts skip connection between the layers in VGG base network and prediction layers. Conv4_1, which undergoes convolutional and pooling layers, is then merged with Conv4_3 to generate Conv4_3_ff, and the latter fc7, Conv6_2, Conv7_2, Conv8_2, and Conv9_2 are merged with their corresponding basic feature layers in a similar way. The fusion feature maps Conv4_3_ff, fc7_ff, Conv6_2_ff, Conv7_2_ff, Conv8_2_ff, and Conv9_2_ff act as the multiscale prediction feature maps.

**Figure 7:**
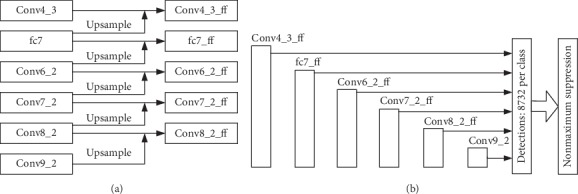
Multiscale prediction feature maps adjacent connection (adjacentSSD). (a) Multiscale prediction feature maps adjacent connection structure. (b) Multiscale prediction layers.

The structure in Figure 7(a) is called AdjacentSSD. Compared with SKIPSSD, AdjacentSSD conducts adjacent connection instead of skip connection. Layer fc7, which undergoes the upsampling layer, is merged with Conv4_3 to generate Conv4_3_ff, and the latter layers Conv6_2, Conv7_2, Conv8_2, and Conv9_2 are merged with their adjacent layers in a similar way. The Figure 7(b) shows that AdjacentSSD uses Conv4_3_ff, fc7_ff, Conv6_2_ff, Conv7_2_ff, Conv8_2_ff, and Conv9_2 as the multiscale prediction feature maps.

**Figure 8:**
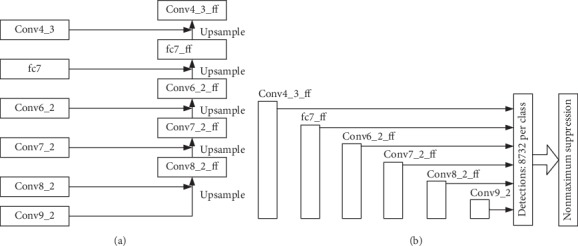
Multiscale prediction feature pyramid network (FPNSSD). (a) Multiscale prediction feature pyramid network structure. (b) Multiscale prediction layers.

**Figure 9:**
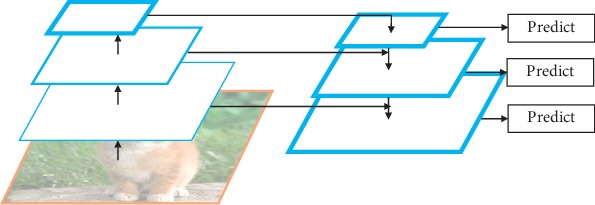
Feature pyramid network.

The structure in Figure 8(a) is called FPNSSD. Different from AdjacentSSD, in which the high-level layer Conv9_2 is only fused with adjacent layer Conv8_2 to generate Conv8_2_ff, FPNSSD adopts a top-down architecture with lateral connections called FPN [16], shown in Figure 9, and the high-level layer Conv9_2 is fused layer by layer and contributes to the generation of Con8_2_ff, Conv7_2_ff, and so on. The Figure 8(b) shows that FPNSSD uses Conv4_3_ff, fc7_ff, Conv6_2_ff, Conv7_2_ff, Conv8_2_ff, and Conv9_2 as the multiscale prediction feature maps.

The abovementioned six multiscale feature maps fusion structures over SSD are analysed and evaluated on the PASCAL VOC2007 test set in Section 3.4 to explore the optimal multiscale feature maps fusion structure.

### 2.3. Feature Fusion Module Design

In this paper, two feature fusion modules are designed for high-level and low-level feature fusion, and the fusion effect is compared in our experiments. Concat and eltsum are two common methods of feature fusion. Concat operation is channel concatenation of two feature maps. In Caffe, there are three operations of the Eltwise layer: product (dot product), sum (add or subtract), and max (take the large value), and the sum operation is selected in this paper.

**Figure 10:**
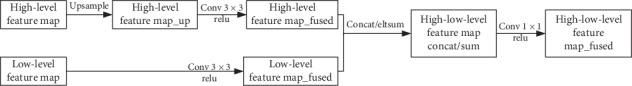
Fusion module *a*.

As shown in Figure 10, fusion module *a* firstly upsamples the high-level feature map to generate high-level feature map_up, which undergoes 3 × 3 convolutional layer and relu activation function to obtain high-level feature map_fused. In addition, the low-level feature map, which undergoes 3 × 3 convolutional layer and relu activation function, is transformed into low-level feature map_fused. Then, concat or eltsum function is applied to conduct feature fusion between low-level feature map_fused and high-level feature map_fused to obtain the high-low-level feature map concat/sum. Finally, the high-low-level feature map concat/sum, which undergoes a 1 × 1 convolutional layer to reduce channel dimensions, is activated with the relu activation function to obtain the prediction feature map high-low-level feature map_fused.

**Figure 11:**
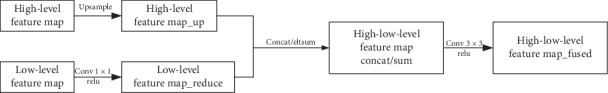
Fusion module *b*.

Compared with fusion module *a* in Figure 10, the fusion module *b* in Figure 11 is briefer. Firstly, fusion module *b* upsamples the high-level feature map to generate high-level feature map_up and transform low-level feature map into low-level feature map_reduce through a 1 × 1 convolutional layer. Then, concat or eltsum function is applied to conduct feature fusion between low-level feature map_reduce and high-level feature map_up to obtain the high-low-level feature map concat/sum. Finally, the high-low-level feature map concat/sum, which undergoes a 3 × 3 convolutional layer to reduce the aliasing effect, is activated with the relu activation function to obtain the prediction feature map high-low-level feature map_fused.

## 3. Results and Discussion

To evaluate the performance of the proposed improved SSD network and to find the optimal multiscale feature maps fusion framework, four types of test cases are designed in this paper:

1. Compare the performance of SKIPSSD when using different feature fusion modules. Through this experiment, the most effective feature fusion module can be found.
2. Compare the influence of different fusion strategies on SKIPSSD model performance. Through this experiment, the most effective fusion strategy can be selected.
3. Compare the effect of different upsampling methods on SKIPSSD model performance. Through this experiment, the most effective upsampling method can be selected.
4. Compare the performance of SSD with different feature maps fusion structures. Through this experiment, the most effective feature fusion structure can be found.

The experimental hardware and software configurations are listed in Table 1. In order to evaluate the performance of SKIPSSD, the union of VOC2007 *trainval* and VOC2012 *trainval* is used as the training data, and the VOC2007 test as the test data. For fair comparison, the experiments are all based on VGG16, which is preprocessed as what is conducted in SSD, and SKIPSSD is trained in the same way as SSD. The parameter settings are listed in Table 2. The mAP and FPS are adopted as the metric for evaluating detection performance.

### 3.1. The Effect of Two Feature Fusion Modules on Model Performance

In order to find the optimal feature fusion module, SKIPSSD with different feature fusion modules are evaluated on the PASCAL VOC 2007 test, and the performance evaluated with an input size 300 × 300 is recorded in Table 3. In this experiment, the network of SKIPSSD is shown in Figure 3, and the BN (Batch Normalization) layer is added after all the convolutional kernels in the fusion module.

According to the results in Table 3, SKIPSSD with fusion module *a* achieves 78.1% mAP, 0.9% higher than SSD, and the mAP of SKIPSSD with fusion module *b* is 1.1% mAP higher than SSD, demonstrating that skip connection of multiscale feature maps indeed improves the performance of SSD. Since SKIPSSD with fusion module *b* outperforms fusion module *a* on both accuracy and speed, the fusion module *b* is chosen in this paper for high-low level feature fusion.

### 3.2. The Effect of Fusion Strategies on Model Performance

In this experiment, two aspects of the factors are compared: (1) concat and eltsum fusion methods; (2) full and partial use of BN layers. The experimental results are recorded in Table 4. In the experiment, the upsampling method is deconvolution and dilated convolution, and the fusion module is *b*.

From the data of the first and third rows in Table 4, it could be concluded that in the same network structure, the eltsum fusion method provides better accuracy than concat does. Comparing the second, third, and fourth rows in Table 4, adding BN layers can improve the accuracy of SKIPSSD. When only using BN layers after the eltsum function, SKIPSSD achieves 78.4% mAP, 0.1% higher than SKIPSSD using BN layers after all convolution layers of the fusion modules. After comprehensive analysis, the SKIPSSD in this paper adopts the eltsum fusion method, and only uses BN layers in the convolution layer behind eltsum function.

### 3.3. The Effect of the Upsampling Method on Model Performance

**Figure 12:**
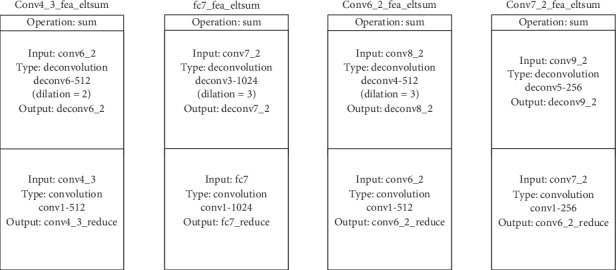
Configuration of sampling parameters on deconvolution and dilated convolution.

**Figure 13:**
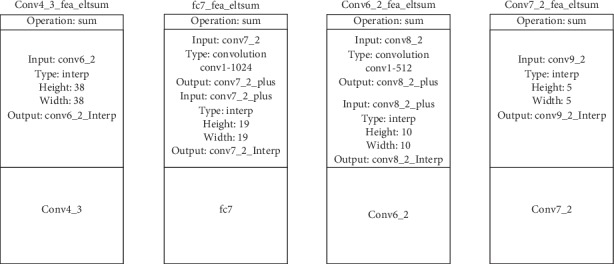
Configuration of sampling parameters on bilinear interpolation.

In order to study the effect of upsampling methods on the performance of SKIPSSD, we adopt two kinds of upsampling methods to feature fusion module of SKIPSSD. The first method is deconvolution and dilated convolution, and the specific network structure parameters are shown in Figure 12. The second method is bilinear interpolation, and the specific network structure parameters are shown in Figure 13.

Table 5 shows that SKIPSSD with the bilinear interpolation upsampling method achieves 79.0% mAP at 38.7 FPS on the PASCAL VOC2007 test set, outperforming the deconvolution and dilated convolution upsampling method both on speed and accuracy. Therefore, bilinear interpolation is selected as the upsampling method in this paper.

### 3.4. Effect of the Feature Fusion Structure on Model Performance

This experiment compares the performance on the PASCAL VOC 2007 test set of six different feature fusion structures: SKIPSSD, Part-SKIPSSD, Bi-SKIPSSD, Base-SKIPSSD, AdjacentSSD, and FPNSSD. In this experiment, the upsampling method is bilinear interpolation, the fusion module is *b*, the fusion method is eltsum, and BN layers are only used in the convolutional layer after eltsum function.

As shown in Table 6, Base-SKIPSSD achieves 78.6% mAP, 1.4% higher than SSD by conducting skip connection between layers of VGG base network and prediction layers. However, the lower prediction layers such as Conv4_3_ff still lack enough semantic information for small object detection. FPNSSD adopts a top-down architecture with lateral connections to build high-level semantic feature maps at all scales which is good for multiscale object detection, but fusing features layer by layer is not efficient enough while there are many layers to be combined together. And AdjacentSSD achieves almost the same performance as FPNSSD, indicating that there is no need to densely fuse features layer by layer via a top-down architecture. Compared with FPNSSD, by fusing low-level and high-level feature maps skippingly, SKIPSSD is more lightweight and efficient. And the performance of Part-SKIPSSD and Bi-SKIPSSD demonstrates that less skip connection do not bring obvious advantage of speed, and more skip connections would cause redundancy and do not bring significant accuracy improvement. Thus, in the end, SKIPSSD network is selected as the optimal feature fusion structure.

### 3.5. Experiments on PASCAL VOC 2007

**Figure 14:**
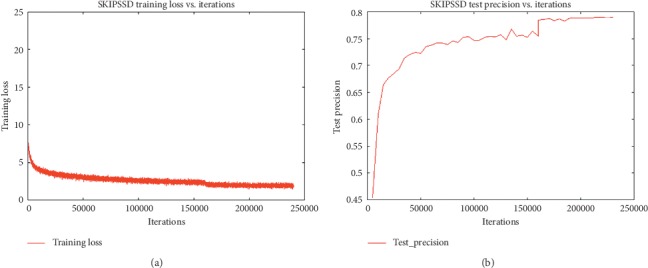
The loss and precision curves. (a) Iterations-training loss curve. (b) Iterations-test precision curve.

The loss curve of SKIPSSD is shown in Figure 14(a). Loss keeps decreasing during the training process. In the first 50,000 steps, the loss decreases sharply. After 16,000 steps, the decline speed slows down further, and the loss curve tends to remain unchanged after 200,000 steps. Accordingly, as shown in Figure 14(b), in the first 50,000 steps, the accuracy increases sharply and tends to remain unchanged after 2000 steps, reaching 79.0% at 22,500 steps.

Table 7 shows the object detection results on the PASCAL VOC 2007 test set. Compared with SSD, SKIPSSD shows a large improvement for 18 classes, including small objects like bottle, boat, bird, plant, and so on, demonstrating that the weakness of small object detection in SSD is improved. With low dimension input 300 × 300, SKIPSSD achieves 79.0% mAP without bells and whistles, outperforming a lot of state-of-the-art object detection algorithms like Faster R-CNN [3], YOLOv2 [18], YOLOv3 [19], and DSSD [10]. Although the mAP of SKIPSSD is 1% lower than RefineDet320 [14], RefineDet_SKIP320 achieves 0.4% mAP higher than RefineDet320 by adopting skip connection of multiscale feature maps, demonstrating that the skip connection proposed in this paper is effective and can also be integrated into other object detectors.

### 3.6. Inference Time

Table 8 shows the comparison of speed and accuracy of SKIPSSD and the state-of-the-art object detectors on the PASCAL VOC 2007 test set. For fair comparison, we also test SSD300 [6], RSSD300 [12], and RefineDet320 [14] on the GeForce GTX 1080.

On a single 1080 GPU, SKIPSSD300 achieves 79.0% mAP at 38.7 FPS, 1.8% mAP higher than the original SSD and surpassing most of the other state-of-the-art object detection models including two-stage and one-stage methods and other improved SSD models. Although the detection speed of SKIPSSD is a bit slower than SSD due to the extra feature fusion between high-level and low-level features, it is still faster than RSSD [12] and RefineDet [14] and is able to realize real-time detection. And RefineDet_SKIP outperforms RefineDet [14] on both accuracy and speed, demonstrating that skip connection proposed in the paper works better than FPN in the object detection task.

### 3.7. Visualization

**Figure 15:**
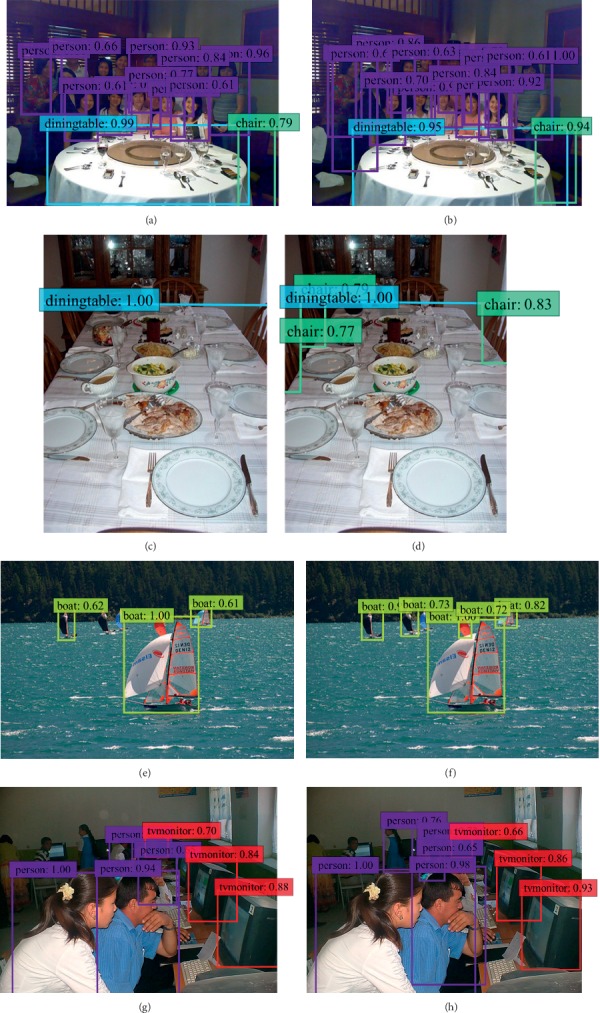
Comparison of detection performances of SSD and SKIPSSD models on some test samples. The first column shows the results of SSD, and the second column is the results of SKIPSSD.

As shown in Figure 15, compared with Figures 15(a) and 15(b), SKIPSSD detects more targets of the same class than SSD when the targets are dense. Compared with Figures 15(c)–15(h), SKIPSSD can detect small objects better than original SSD, and can also “capture” distant objects, which proves that the proposed SKIPSSD based on skip connection of multiscale feature maps can improve the performance of the whole model and the detection performance of small objects.

## 4. Conclusions

In this paper, an improved SSD algorithm SKIPSSD based on skip connection of multiscale feature maps is proposed. In order to fuse high-level and low-level features effectively, a variety of feature fusion modules and fusion connection modules are designed and compared. Experimental results show that with an input size 300 × 300 on 1080 GPU, SKIPSSD achieves 79.0% mAP at 38.7 FPS, 1.8% higher than SSD and can still keep real-time detection speed. In addition, although the skip connection is only adopted to SSD and RefineDet in this paper, it can also be integrated into other object detectors.

In the future work, channel attention mechanism will be adopted to filter out the unimportant channels and improve the saliency of features by learning the importance of each channel.

## Figures and Tables

**Table 1:** Experimental hardware and software configurations.

Hardware and software	Profile
CPU	Intel XeonE5-2620v4@2.10GHz
GPU	GeForce GTX 1080
Operating system	Ubuntu 16.04
Deep learning frame	Caffe

**Table 2:** Training parameter settings.

Parameter	Size
Input size	300 × 300
Number of iterations	240,000 steps
Batch size	16
Initial learning rate	0.0005 (it is divided by 10 in steps of 160,000, 200000, and 240,000)
Weight learning rate	0.0005
Momentum	0.9

**Table 3:** The effect of the feature fusion connection module on model performance.

Model	Data	Pretrained model	Fusion module	Fusion method	FPS	mAP (%)
SSD	07 + 12	VGGNet	×	×	41.4	77.2
SKIPSSD	07 + 12	VGGNet	*a*	Eltsum	32.3	78.1
SKIPSSD	07 + 12	VGGNet	*b*	Eltsum	37.8	78.3

**Table 4:** The effect of different fusion strategies on model performance.

Model	Data	Pretrained model	Fusion model	BN	mAP (%)
SKIPSSD	07 + 12	VGGNet	Concat	Used after all convolution layers of the fusion module	77.9
SKIPSSD	07 + 12	VGGNet	Eltsum	×	78.0
SKIPSSD	07 + 12	VGGNet	Eltsum	Used after all convolution layers of the fusion module	78.3
SKIPSSD	07 + 12	VGGNet	Eltsum	Used only after the eltsum function	78.4

**Table 5:** Effect of different upsampling methods on model performance.

Model	Data	Pretrained model	Upsampling method	FPS	mAP (%)
SKIPSSD	07 + 12	VGGNet	Deconvolution + dilated convolution	36.8	78.4
SKIPSSD	07 + 12	VGGNet	Bilinear interpolation	38.7	79.0

**Table 6:** Effect of different feature fusion network structures on model performance.

Model	Data	Pretrained model	FPS	mAP (%)
SKIPSSD	07 + 12	VGGNet	38.7	79.0
Part-SKIPSSD	07 + 12	VGGNet	39.2	78.7
Bi-SKIPSSD	07 + 12	VGGNet	38.1	78.8
Base-SKIPSSD	07 + 12	VGGNet	39.2	78.6
AdjacentSSD	07 + 12	VGGNet	38.0	78.8
FPNSSD	07 + 12	VGGNet	37.9	78.8

**Table 7:** Object detection results on the PASCAL VOC 2007 test set.

Method	mAP	Aero	Bike	Bird	Boat	Bottle	Bus	Car	Cat	Chair	Cow	Table	Dog	Horse	Mbike	Person	Plant	Sheep	Sofa	Train	Tv
Fast [2]	70.0	77.0	78.1	69.3	59.4	38.3	81.6	78.6	86.7	42.8	78.8	68.9	84.7	82.0	76.6	69.9	31.8	70.1	74.8	80.4	70.4
Faster [3]	73.2	76.5	79.0	70.9	65.5	52.1	83.1	84.7	86.4	52.0	81.9	65.7	84.8	84.6	77.5	76.7	38.8	73.6	73.9	83.0	72.6
YOLOv2 416 [18]	76.8	87.9	87.5	78.2	61.5	57.9	84.9	82.9	90.6	54.9	83.6	66.5	90.1	85.2	85.8	82.9	54.2	78.9	65.2	87.3	69.8
YOLOv3 416 [19]	78.3	88.7	84.3	76.1	67.6	62.8	85.7	88.8	88.9	60.4	83.6	71.6	86.0	87.9	86.4	81.7	49.1	81.1	76.6	84.9	74.7
SSD300 [6]	77.2	79.2	83.5	75.7	70.0	51.0	86.7	86.0	86.8	60.1	80.9	76.8	85.8	85.8	84.2	79.6	52.7	78.6	77.4	86.9	77.3
DSSD321 [10]	78.6	81.9	84.9	80.5	68.4	53.9	85.6	86.2	88.9	61.1	83.5	78.7	86.7	88.7	86.7	79.7	51.7	78.0	80.9	87.2	79.4
Feature-fused SSD [13]	78.9	82.0	86.5	78.0	71.7	52.9	86.6	86.9	88.3	63.2	83.0	76.8	86.1	88.5	87.5	80.4	53.9	80.6	79.5	88.2	77.9
RefineDet320 [14]	80.0	83.9	85.4	81.4	75.5	60.2	86.4	88.1	89.1	62.7	83.9	77.0	85.4	87.1	86.7	82.6	55.3	82.7	78.5	88.1	79.4
SKIPSSD300	**79.0**	**82.5**	**85.1**	**78.8**	**73.0**	**51.2**	86.6	**87.0**	**89.2**	**63.8**	**85.2**	**77.8**	**87.0**	**87.3**	**86.0**	79.4	**53.4**	**79.1**	**79.8**	**88.0**	**79.3**
RefineDet_SKIP320	80.4	83.3	85.3	79.5	74.2	60.9	87.8	88.3	87.9	65.8	85.8	77.5	85.3	87.5	86.4	83.6	57.0	81.5	80.2	88.4	81.2

**Table 8:** Comparison of speed and accuracy on the PASCAL VOC2007 test dataset.

Model	Data	Base network	mAP (%)	FPS	GPU	Input size
Faster R-CNN [3]	07 + 12	VGGNet	73.2	7	Titan X	∼600 × 1000
R-FCN [4]	07 + 12	ResNet-101	79.5	9	Titan X	∼600 × 1000
YOLOv2 [18]	07 + 12	VGGNet	76.8	67	Titan X	416 × 416
YOLOv3 [19]	07 + 12	VGGNet	78.3	57.7	Titan Xp	416 × 416
SSD300 [6]	07 + 12	VGGNet	77.2	46	Titan X	300 × 300
DSSD321 [10]	07 + 12	ResNet-101	78.6	9.5	Titan X	321 × 321
DSOD300 [20]	07 + 12	DS/64-192-48-1	77.7	17.4	Titan X	300 × 300
RSSD300 [12]	07 + 12	VGGNet	78.5	35	Titan X	300 × 300
FSSD300 [9]	07 + 12	VGGNet	78.8	35	Titan X	300 × 300
RefineDet [14]	07 + 12	VGGNet	80.0	40.3	Titan X	320 × 320
SSD300 [6]	07 + 12	VGGNet	77.2	41.4	1080	300 × 300
RSSD300 [12]	07 + 12	VGGNet	78.5	34.8	1080	300 × 300
RefineDet [14]	07 + 12	VGGNet	80.0	36.0	1080	320 × 320
SKIPSSD300	07 + 12	VGGNet	79.0	38.7	1080	300 × 300
RefineDet_SKIP	07 + 12	VGGNet	80.4	37.0	1080	320 × 320

## Data Availability

The data used to support the findings of this study are available from the corresponding author upon request.
